# Weighing heavy: Heavy serving dishes increase food serving

**DOI:** 10.1371/journal.pone.0288956

**Published:** 2023-08-25

**Authors:** Aner Tal, Amir Grinstein, Mirella Kleijnen

**Affiliations:** 1 College of Law and Business, Ramat-Gan, Israel; 2 Northeastern University, Boston, Massachusetts, United States of America; 3 Vrije Universiteit Amsterdam, Amsterdam, The Netherlands; University of Canberra, AUSTRALIA

## Abstract

The current work demonstrates that people serve themselves greater amounts of food when carrying heavier serving dishes. This effect occurs because increases in carried weight lower consumers’ sensitivity to the weight of the food served. Decreased sensitivity to weight of food served in turn leads people to continue serving past the point where they would normally stop. The paper demonstrates this effect across two lab studies involving actual food serving (with a third lab study extending the outcomes to unhealthy food choices reported in the S1 Appendix). The studies also demonstrate liking for the food moderates the effect, such that carrying greater weight leads people to serve an increased amount of liked, but not of less well liked, foods. The findings extend prior research regarding the effects of dish weight on food judgment to provide a first demonstration of effects of weight not only on judgment but on behavior. In this, they help expand our understanding of the ways in which elements in the eating environment effects food consumption. In addition, the studies provide initial evidence for the mechanism behind the phenomenon: reduced sensitivity to weight. The research carries important implications for public well being, given that increases in serving sizes may contribute to obesity.

## 1. Introduction

Can the weight of serving dishes effect how much food people serve? Given the prevalence of obesity [1, 2], and its potential negative health consequences [3, 4], reducing overserving may be beneficial to consumers. Considerable overserving is generated by environmental influences [5] that contribute to eating beyond physical needs [6]. Still, the environment can serve to curb overeating and promote healthier eating behavior [7].

The current work focuses on the effects of one understudied environmental factor that may contribute to overeating: the weight of serving dishes such as trays, plates, and other dishes consumers use to serve themselves food. The paper suggests that greater weight can lead to increased food serving. Specifically, building on psychophysical theories regarding sensitivity to physical properties, this research argues that carrying an increased base weight reduces sensitivity to additional weight. Due to lower sensitivity to the amount of food they serve, consumers would notice having served “enough” later on, and consequently serve greater amounts of food. In other words, reduced sensitivity would tend to lead to a bias in amount served as well as estimations of amount served.

A few recent studies have supported the idea that weight cues can contribute to food evaluation [8–12]. However, these studies all focused on food judgment and consumption experience—i.e., taste, perceived quality—while not addressing the impact of weight on food serving and consumption. The current research contributes to this literature by examining the effects of weight on serving, as well as extending our understanding of the impact of weight carried by providing evidence for weight sensitivity as the mechanism behind the phenomenon.

From a theoretical perspective, the current research could advance understanding of how contextual factors shape food serving, examining the involvement of psychophysical processes, and specifically of weight carried while serving food. Notably, the effects of serving dish weight on food serving has not yet been examined. The current research demonstrates that weight can affect not just food evaluation, but estimation of quantity. Further, the current studies utilized new forms of weight unexplored by previous research: trays. In addition, the paper identifies liking for the food as a key moderating condition for the effects of weight.

Better understanding of the influence of weight of the serving dish on food serving suggests an easy intervention or nudge to alter how much food people serve themselves. Specifically, overeating could be attenuated by providing lighter dishes in both public and home settings.

The paper is organized as follows. First, the paper reviews some of the evidence concerning environmental factors that lead to unhealthy food serving and increased food consumption. Next, the paper elaborates on the effects of weight in consumer judgment. We then discuss how in addition to its effects on product judgment, weight carried can have an impact on food serving through the psychological mechanism of reduced sensitivity to weight. The paper then presents two studies showing that tray weight influences food serving, as well as the underlying process and a moderating condition. We also report another study in the S1 Appendix to extend our findings to unhealthy choices. All evidence is obtained from actual food serving studies, increasing its ecological validity and relevance. Following the presentation of the studies, the paper discusses the theoretical, policy, and practical implications of the current findings, as well as limitations and possibilities for future research.

### 1.1 Effects of environment on eating

Eating behavior is often guided by a variety of environmental cues, which influence food serving as well as consumption quantity [13, 14]. Consumers are influenced by these without necessarily being aware of their effects. In general, food consumption is often determined by elements in the physical and social environment rather than by conscious deliberation [5, 15, 16]. Prior research has shown that a variety of elements in consumers’ environment lead to increased food servings and consumption [6, 17].

One major factor influencing how much people eat is the amount of food they have on their plates. Portion sizes have increased over the years, meaning that more food is available during meals [6, 17]. Greater portions in turn lead to increased consumption [18–20]. A meta-analysis has confirmed widespread effects of portion size on food consumption, indicating that doubling of portions results in a 35% increase in consumption [21]. The increased consumption following increased portion sizes in turn contributes to increases in obesity [22–24].

Other cues in consumers’ environment exert a prominent role in regulating and guiding consumption [6, 25]. For example, larger plates may at times lead to increased consumption. People interpret the size of the plate or the bowl as a cue to the amount of food they should serve themselves, such that a larger plate may lead to serving and eating more food, though findings in this area are mixed [26–28].

Similar to plate size, the size of food packages can also exert an influence on amount eaten. Consumers may continue eating as long as food remains in a package, such that the more food there is in a package, the more they eat. Larger package sizes have accordingly been linked to increased consumption [20], while smaller package sizes can lead to decreased consumption [29–31].

In sum, prior research indicates that elements in one’s environment, including packages, plates, and utensils, may all influence the amount of food served and eaten. The current work expands this research to examine how the weight carried by consumers, with a focus on weight of the serving dishes—can serve as another environmental cue that effects food serving and consumption.

### 1.2. Weight of dishes and food evaluation

Extensive research examines the influence of sensory characteristics of food products on consumer judgment. Sensory characteristics going beyond both the product itself and peripheral elements such as its packaging can have extensive influence on product evaluation [32, 33].

Stimuli related to weight of the food has come under growing attention in the past decade. Weight of food packaging, such as bottles, has been shown to affect judgment of drink price and quality, with heavier bottles seen as more expensive, and accordingly of greater quality [10]. Similar findings were found with serving cups, with beverages served in heavier cups being perceived as having more intense flavor [11] for some drink flavors. Similarly, the weight of food packaging has been shown to influence judgment of food tastiness, with heavier packages leading to increased judgment of quality, as well as intensity of flavor [34]. Additional research has shown that heavier container weights alters evaluation of food properties related to satiety. Namely, foods in heavier containers are expected to be more satiating [35] and foods served in heavier dishes are seen as more dense, and consequently more filling [9]. The weight of containers can also influence volume estimation, preventing the occurrence of the elongation bias [36].

The weight of elements that are peripheral to the food itself has also been shown to affect food evaluation. For example, restaurants with heavier menus are perceived as more upscale and possessing better service [37]. The weight of serving dishes specifically [e.g., serving plates and cutlery] has been shown to affect judgment of food taste and quality [12, 38]. For example, eating yoghurt with heavier spoons leads consumers to rate the yoghurt as tastier [8]. Similar effects have been found in field settings, with diners using heavier cutlery at a restaurant reporting enhanced experience and evaluation of the food than those using lighter cutlery [12].

The weight of serving dishes has been shown to effect evaluation of attributes going beyond quality and taste as well. For example, lighter food is seen as healthier [39, 40]. Even symbolic representations of weight, such as with chunkier fonts, can map on to perceptions of healthiness, with consumers rating food in packaging with “heavier” fonts as less healthy [39]. Such effects of lightness in fact extend to symbolic representations of lightness. For example, lighter colors on packaging as well as lighter color saturation lead to evaluation of food as healthier [41, 42]. This is because lightness of color, as well as actual physical lightness, is associated with healthiness [43].

The effects of weight on food evaluation have been explained by learned associations. For example, heavier menu weights are associated with greater quality of a restaurant [37]. Similarly, lighter weights are associated with other attributes such as quality and healthiness in consumers’ minds, so that encountering lightness leads consumers to deduce reduced quality [10] or greater healthiness [43, 44]. This is true for both physical lightness and symbolic representations of lightness, as with light colors [43]. Such effects can be explained as a case of sensation transference, cross modal effects where the haptic attributes of the food or surrounding stimuli such as packaging or serving dishes translate to evaluation of the food on dimensions such as tastiness, healthiness, and other attributes of the food [8, 9, 34]. Consumers transfer properties of the serving dish or utensil to judgment of properties of the food.

While considerable research has examined the impact of weight on food evaluation, to the best of our knowledge no research to date has examined the effects of serving dish weight on the amount of food served, a crucial variable given its potential to lead to overeating. Next, we therefore expand the literature regarding the effects of weight to look at the effects of weight on the amount of food served, rather than just food judgment.

#### 1.2.1 Sensitivity to weight: A psychophysical explanation

Sensory adaptation is a process by which initial sensory experiences with a stimulus change sensitivity to subsequent sensory stimulus [45, 46]. People become accustomed to a particular level of stimulus property, and become desensitized to it. Reduced sensitivity may in turn reduce awareness to changes in the stimulus. If the base level of a physical stimulus is high and a person adapts to it, they may become desensitized to additions in the stimulus, such that a greater change in the level of the stimuli would be required in order to be noticed [47, 48]. For example, increased sweetness levels desensitize to further sweetness [49], and with respect to weight, carrying and being adapted to higher weight could change one’s sensitivity to additional weight. In the current context, higher base weight of serving dishes would impact one’s sensitivity to additional weight given by food carried.

Weber’s Law offers a related but distinct phenomenon whereby sensitivity to additional weight is dependent on current carried weight [50–52]. Weight is judged in relation to a baseline, such that people are less sensitive to additional weight the higher the base weight they carry [53]. In fact, a change of approximately 10% of the base weight is needed to detect change [54]. This would lead people to notice additional weight to a lesser extent.

Reduced sensitivity to weight could, in the context of food servings, lead people to be less sensitive to the amount of food they serve themselves. The heavier the weight people carry in the form of a serving dish, the less they would notice differences in the amount of food they serve, and the higher the change in the amount of food served needed in order to be noticed. This is because the ability to detect differences in weight served is proportionate to the baseline weight carried. This would lead people to notice having served a sufficient amount of food later on in the process of serving, consequently serving themselves a greater amount of food. Further, because the weight is noticed less, people will likely underestimate the amount of food they are serving as well as the weight of the food served. This is because when carrying a heavier base weight, the weight food served is noticed less, and therefore estimated as lower. In sum, reduced awareness of the amount of food served may lead people to register a lower amount of food served, and consequently serve themselves more food before they register that they have enough.

### 1.3 Summary and hypotheses

To date, research regarding the effects of weight in the food domain focused on judgment, indicating that greater weight of dishes alters food evaluation [8, 10, 12, 34]. The current research contributes to this literature by providing a first demonstration of the effects of weight on behavior. Specifically, based on the logic above, we argue that increased weight of serving dishes leads people to be less sensitive to the weight of the food served on to serving dishes. This in turn leads them to require greater amounts of food in order to register that they’ve served themselves enough. This is because due to reduced sensitivity to weight, more food is required to feel that enough food has been served. Formally, we predict:

**H**_**1**_: **Heavier serving dish weight leads consumers to serve themselves a greater amount of food**.

Further, in support of our explanation regarding reduced sensitivity to weight served, we predict lower estimates of the weight of food served when carrying a heavier weight. If carrying a heavy tray leads to reduced sensitivity to weight, consumers should not only serve themselves more food, but in addition, conversely, judge that they have served themselves relatively less food. This is because, if our explanation is correct, the base weight carried should reduce sensitivity to the weight of the food served, such that the food served is noticed to lesser extent. Consequently, people would consider that they have served themselves less weight when holding a higher base weight. In other words, given that weight is sensed relative to a baseline, consumers would estimate lower weight of the food they served when carrying a heavier tray.

**H**_**2**_: **Heavier weight of the serving dish leads consumers to estimate lower amounts of food served**.

The paper tests these hypotheses across two studies that examine actual food serving. In addition, a third study that extend our findings to unhealthy eating as well is included in the S1 Appendix.

## 2. Materials and methods

### 2.1 Study 1: Heavier trays lead to increased food serving

The current study was designed to provide an initial test for the primary hypothesis. The study aimed to examine whether carrying heavier trays would lead to increased serving of food (H_1_). For that purpose, the study compared the amount of food served by participants randomly assigned to carry either a regular or weighted tray while serving themselves food.

#### 2.1.1 Methods

Fifty one participants (30 female, 2 unrecorded) completed a two-cell (tray: light, heavy) IRB-approved study for credit (Protocol AG04122012, Ben Gurion University, and protocol 110-100-1899, Cornell University, were used for all studies). Participants were over 18 years old and completed written informed consent forms. Participants completed the study in groups of 4 to 8 participants. Each group was randomly assigned to an experimental condition (light vs. heavy trays). Light trays weighed 495 grams and heavy trays weighed 705 grams. Heavy trays were modified by gluing a layer of heavy rubber to the bottom of the tray. Given that the tray was approximately 200 grams heavier, the just noticeable difference should be one in excess of 20 grams. This is in line with Weber’s Law dictum of approximately 1/10^th^ of the base weight being the just noticeable difference [54]. In other words, with the trays being 200 grams heavier, participants should be able to serve themselves up to 20 more grams without realizing they’d served extra.

Participants completed the study at the end of a one hour study session comprising unrelated studies, in which they had eaten other snacks. However, snacks given prior to the study were small (a bite-size cookie or chocolate) and so would have only whetted participants’ appetites rather than satiated them. Participants were not given information about the purpose of the study and were merely informed it was a food study. Experimenters conducting the study were left uninformed about the purpose of the study, to avoid biasing results.

Each participant was given a tray and were told they could serve themselves baby carrots (healthy option) and Chex Mix (unhealthy option), and that they could serve as much or as little of each as they wished. Researchers recorded the weight served of each of the two snacks. To ensure weight was felt during serving, participants were asked to hold the tray as they served themselves. Experimenters were present to make sure participants did indeed keep holding the tray while serving. The serving ladles were large enough that most participants could serve themselves as much Chex mix and baby carrots as they wanted using it only once, such that the duration of holding the tray during serving would not change radically between participants. The serving bowl was refilled throughout the study between experimental groups to retain a consistent level of the snack. The snacks were served onto paper bowls placed on each participant’s tray. The bowls were light paper bowls (10 grams), chosen so as not to radically alter the tray weight.

Participants were asked to serve only as much Chex Mix and baby carrots as they actually wanted to eat. They were also informed that they would be required to leave the bowl in the lab and would therefore need to finish eating what they served before leaving. This was done to try to ensure that participants served an amount they actually wanted to consume.

After serving themselves as much Chex Mix and baby carrots as they desired and before consuming the food, participants handed their bowls to an experimenter for weighing. Results and Discussion.

The analysis (F-tests within a mixed model with repeated measures) examined the weight of Chex Mix and baby carrots served by participants and found directional increases in amounts served of both snacks. Means and standard deviations are presented in Table 1. We tested effects of tray weight on amount served using a mixed model with snack type as a repeated measure The covariance structure was specified as unstructured, for minimal assumptions. Within the mixed model, t the effect of weight on amount served was tested using an F test. There was a significant effect of tray weight on food serving: F(1, 49) = 6.16, p = .02.

**Table 1:** Serving of Chex Mix versus baby carrots (Study 1).

Grams served Means (SD)	Light	Heavy
Chex Mix	28.63 (19.06)	40.82 (16.04)
Baby Carrots	77.96 (47.33)	92.53 (72.54)

Next, analysis was conducted to examine whether or not effects on serving Chex Mix and baby carrots differ. Accordingly, an interaction term was added to the model described above. In other words, the model now included tray weight, food type, and the interaction of the two. The analysis revealed no significant interaction effects: F(1, 49) = .03, p > .1. In other words, the effects of tray weight on food serving did not differ between food items.

It appears that increased tray weight leads to increases in amount of snacks served (H_1_). This result supports the idea that weight of the tray leads to increased serving and in turn consumption. The next study was intended to examine whether or not the effects of heavier trays do indeed lead to reduced sensitivity to weight of food served, and whether or not the effect is moderated by liking for the food served.

### 2.2 Study 2: Replication, moderating condition, and underlying process

Study 1 showed that heavier trays lead consumers to serve themselves more food. The study aims to replicate the effect found in the first study, and further study the potential process and a moderating condition by gathering evidence supporting reduced sensitivity to weight given heavier tray weight, as well as moderation of the effects of weight on food serving by liking.

Indeed, the study was designed to provide evidence for our proposed theoretical explanation by supporting H_2_, arguing that weight of serving dish would lead to estimating lower amounts of food serving. Such a finding would help corroborate reduced sensitivity to weight as the underlying explanation for increased serving when carrying heavier serving dishes.

In addition, the current study aimed to explore liking of the food served as a moderating condition for the effects of weight on food serving. The effects of liking have been examined in research studying the effects of environment on food choice. Prior research demonstrates that the food serving environment has particularly marked effects on consumption of well-liked foods [55, 56]. When food is well-liked, environmental factors influencing consumption produce greater effects than they do for less well-liked foods. Automatic eating might occur more when the food is well-liked [55, 56]. This is because when a food is appealing to a consumer, a conscious act of self-control is required in order to monitor serving and lead consumers to stop serving. Reduced sensitivity and awareness of the amount of food served would hamper the possibility for such self-regulation, and hence potentially lead consumers to serve increased amounts of well-liked foods. Concordant with the moderating role of liking, most environmental effects on eating have been documented with well-liked foods such as salty snacks [56] and candy [57]. In the current context, ignorance of amount served may serve as a convenient “cover” for increased portions, legitimizing serving more food because consumers notice the amount of food served to a lesser extent. Reduced sensitivity to tray weight should increase serving more for desired foods, since consumers would notice that they’ve had enough of the well-liked food and should stop serving later on.

For foods they like less, consumers may not serve as much, as a consequence of reduced weight sensitivity, since their automatic tendency to take more food may be attenuated. If the food is not liked, people may not consume it automatically, such that mindless eating effects would be less pronounced.

In sum, liking for food should serve as a moderating condition for the effects of weight on food serving. Specifically, the effects of heavy trays should be particularly pronounced for likeable foods, where less likeable foods are less likely to be affected by heavier trays. Such an effect would be consistent with prior research documenting greater effects of the environment on eating behavior for well-liked foods [55, 56]. Formally:

**H**_**3**_: **Liking for food would moderate the effects of serving dish weight on amount served**.

#### 2.2.1 Methods

The research team recruited 40 for-credit Undergraduate and Master’s student participants (23 female) from the participant pool of a large Northeastern University. Participants were over 18 years old and completed written informed consent forms. They completed this IRB-approved study as part of a longer session involving multiple unrelated experiments. Participants were randomly assigned to carry either the heavy or regular tray used in the last study.

The procedure used was similar to that used in the previous study, but with Chex Mix only. Each participant was given a tray and instructed to serve as much Chex Mix as they would like to eat from a large serving bowl using a ladle. Both participants and experimenters were not informed on the purpose of the study. Participants were asked to hold the tray as they served themselves, with experimenters present to ensure participants continued holding the trays while serving. The Chex Mix was served onto the same paper bowls used in the previous study. The serving bowl was refilled throughout the study between experimental groups to retain a consistent level of the snack.

Again, participants were asked to serve only as much Chex Mix as they actually wanted to eat, and were informed that they would be required to leave the bowl in the lab, which meant they would need to finish what they served before leaving. This was done to try to ensure that participants served an amount they actually wanted to consume.

Serving amount and estimated amount served were measured similarly to the last experiment. Participants also estimated the amount of food they served themselves.

In addition, participants were asked to report how much they liked Chex Mix on a scale of 1—“not at all” to 9—“very much”. This measure was intended to garner support for H_3_, by showing increased effects of weight carried for those who like Chex Mix.

#### 2.2.2 Results and discussion

Analysis was conducted using ANOVA and ANCOVA tests using GLM on SAS. The analysis began with an ANOVA testing for differences in amount served between the heavy and light tray conditions. F-test revealed a significant increase in Chex Mix served for heavy trays (*M* = 31.19 grams) over light trays (17.71 grams): *F(1*, *36) = 6*.*76*, *η*_*p*_^*2*^ = .16, *p* = .*01*. Carrying a heavier tray led participants to serve themselves almost twice as much Chex Mix, providing support for the tendency to serve more when carrying heavier trays and H_**1**_. Results can be seen in Table 2.

**Table 2:** Serving of Chex Mix (Study 2).

Grams served Means (SD)	Light	Heavy
Chex Mix	27.71 (12.04)	41.19 (18.40)

To further support the idea that the weight of the dish reduces sensitivity to amount served and therefore makes participants notice the amount served to lesser extent, F-test analysis examined the differences in estimated amount served. Contrary to the actual amount served (where heavy tray participants served more), participants in the heavy tray condition estimated serving themselves less food (59.57 grams) than did regular tray participants (91.36 grams). In accordance with the previous analysis in this study, ANCOVA was used to examine the differences in estimated serving. The model included the actual amount served, liking for Chex Mix and its interaction with tray weight. The main effect of weight of the tray on amount served was significant: *F*(1, 33) = 4.73, *η*_*p*_^*2*^ = .11, p = .04. Participants carrying heavier weight served themselves greater amounts of Chex Mix but estimated lower amounts, providing support for H_2_. This supports the role of reduced sensitivity to weight when carrying heavier trays.

Finally, the following analysis examined the influence of liking for Chex Mix on amount served. For this, F-test analysis was conducted using a general linear model with amount served as a function of condition, liking for Chex Mix, and the interaction of the two. Accounting for liking and its interaction with weight of the tray produced a significant interaction but undid the simple effects of weight carried on amount served (*F*(1, 34) = 1.98, *p* = .17), indicating that the effect is contingent on liking for the product. There was a significant interaction of liking and tray weight such that differences in amount served between light and heavy trays were higher for participants who expressed stronger liking for Chex Mix. The interaction was significant at a the .02 level: *F*(1, 34) = 6.14, *η*_*p*_^*2*^ = .11. There was no main effect of liking (*p* = .26, *η*_*p*_^*2*^ = .02). The significant interaction between tray weight and liking lends support to H_**3**_.

In summary, as in study 1, participants holding heavier trays served themselves more food than participants holding lighter trays. New to study 2, heavier trays produced relatively lower estimates of the amount of food served, consistent with the prediction of reduced sensitivity to weight, while demonstrating that liking of the food enhances the effect of weight carried.

## 3. General discussion

Two studies demonstrated that heavier serving dishes lead consumers to serve themselves greater amounts of food, especially well-liked foods. These effects of heavier dishes appear to occur because the heavier the dish, the less sensitive consumers are to the weight of the food they serve.

In study 1, participants carrying a heavier tray served themselves a greater amount of Chex Mix and baby carrots. Study 2 replicated these effects, demonstrating that heavier trays led to increases in serving Chex Mix. In addition, the study demonstrated that increased serving is moderated by liking for the snack. Finally, the study shows that despite serving themselves more, participants with heavier trays estimated lower weight of the food they served, supporting the notion that weight of the tray reduces sensitivity to the amount of food served.

While a large number of recent studies have examined the impact of environmental cues on food decision-making [e.g., 8, 16, 26, 58], the effects of dinnerware, serving dishes, or other types of physical weights have received less attention in the context of food serving, with most prior work concerning weight focusing on judgment-related outcome of foods.

The moderating role of liking is consistent with the account that eating without awareness is facilitated by reduced sensitivity to cues that could lead consumers to stop serving more food, such as sensing that enough has been served. Indeed, such effects in general occur more for well-liked, tastier foods [55, 59]. Being less sensitive to food quantity may prevent consumers from exerting adequate self-control, as they do not realize how much they have served, which may be a necessary cue to prompt stopping the food serving process, particularly for well-liked foods where self-control is needed. This may constitute a type of (un)willful ignorance [60], such that for well-liked foods, reduced sensitivity to weight may allow consumers to unwittingly ignore their increased level of food serving.

To extend our findings we conducted a third lab study (details reported in the S1 Appendix). For greater generalizability, the study included a different serving dish (heavier vs. standard plate) and a larger set of snack options to choose from (healthy vs. unhealthy options). Participants were asked to choose food items to put on their plate. The results suggested a preference for the unhealthy (more likeable) foods when using the heavier plate. In addition, participants were asked to estimate the calories of the snacks they have put on the plate, and as expected, those using the heavier plate estimated a lower number of calories compared to those using the lighter plate.

### 3.1 Theoretical implications

Positioned against current knowledge regarding the effects of weight in food evaluation [8, 10, 12, 34], the studies offer several important contributions. First, they are the first, to our knowledge, to provide a demonstration of effects of weight on behavior rather than just evaluation. Second, the studies show that weight influences judgment not just of attributes of the food, but of its quantity. Third, the studies offer evidence for psychophysical alterations in sensitivity to weight as a new process involved in the effects of weight on food behavior. Fourth, prior research has demonstrated main effects of weight only. The current research suggests liking of the food as a moderating condition for the effects of weight on food serving. Finally, the studies use trays, going beyond past research examining plates, cutlery, and packaging.

From a theoretical perspective, the studies contribute to several research streams. First, the studies contribute to research on the influence of the environment on eating by documenting an important factor in consumers’ serving environment (serving dish weight) that can trigger increased food serving, particularly of more desirable foods. Notably, the studies address the influence of the environment on food *serving*, demonstrating that consumers may be ignorant or unaware of amount served. Increased serving should in turn translate to eating as well, as consumers tend to consume what they serve [18–20]. This adds to prior research, which has only examined the effects of weight on judgment and evaluation [8, 9, 12, 34, 35].

The nature of the manipulation highlights a key difference from prior work. Specifically, a large body of research considers strategies (e.g., portion size, packaging, display of food servings) that encourages healthier eating using conscious, informational approaches [e.g., 61]. The current research contributes to knowledge of more subtle, less conscious influences impacting food serving and consumption. Our results in study 2 provide evidence that people are not aware of the amount of food served. This lack of awareness regarding food served is important since effects that occur without awareness are harder to control and eliminate.

In addition, the studies suggest a plausible underlying psychological account. The effects of heavier dishes on food serving appear to be due to reduced sensitivity to weight. This constitutes a novel effect of weight of serving dishes on consumer judgment and behavior, going beyond past research implicating sensation transference in the effects of weight on food evaluation [8, 9, 34]. In support of this mechanism, participants in study 2 estimated lower weights (and in the S1 Appendix study lower calories), ostensibly since they were less sensitive to the weight they put on their plates. The size of the effects found lends support to the weight sensitivity explanation, given that Weber’s Law argues changes in weight need to be over 10% of the base weight to be detected [54]. In our case, the additional serving is on average just below such a detection threshold at about 20 grams, relative to 200 grams of added base weight.

Finally, the findings reveal a consistent and interesting interaction between weight and liking. Participants in the current studies served more food when carrying a heavier tray or platter (studies 1–2), but the effect of tray weight on amount served was generally stronger for those who liked the offered snack (study 2).

### 3.2 Policy and practical implications

The current studies have important implications for consumer welfare that are actionable at individual, organizational, as well as policy levels. Across many situations, consumers carry weight (e.g., holding bags, carrying a child, and using platters of varying heaviness) while making food-related decisions. The current findings demonstrate that greater serving dish weight carried may lead to reduced sensitivity to the amount of food they serve, leading to increased food consumption, which may in turn contribute to overeating. Consumers and businesses alike should be made aware that increased tray weight can lead to increased food serving, so that preventative measures can be taken. For example, all-you-can-eat buffets could avoid dishes that are heavier than the norm, thereby reducing over-serving and encouraging healthier portions.

When possible, organizations where consumers serve food may want to opt for lighter trays, plates, and other serving dishes. Organizations that host self-service cafeterias (e.g., companies, government agencies, universities) may benefit from lighter trays or platters. Reducing serving dish weight may be helpful in guiding consumers towards healthier serving sizes.

Consumers themselves often look for strategies to manage food serving situations [62]. The current research may offer another method, or nudge, to help consumers improve eating habits. Specifically, encouraging a greater prevalence of lighter serving dishes in settings where consumers self-serve may help reduce overserving. However, awareness of biasing effects on food serving may not suffice to eliminate them. This is because awareness may not suffice to eliminate unconscious behavioral effects [63]. While awareness may be a good first step in the direction of eliminating the effects it may not suffice to induce behavior change [60, 64].

Policy interventions that attempt to enhance responsible food consumption often involve both regulations and informational campaigns [7, 65]. However, the effectiveness of informational campaigns has come into question [66]. Even with information and knowledge, many consumers lack the motivation or self-control to change [63]. The current research may be effectively used as part of policy tactics that can help nudge consumers towards less automatic food servings by altering the food environment, without need for conscious effort and reflection.

Overall, consumer and industry interests may be aligned in the studied context. Organizations would save money by reducing the amounts consumers serve themselves, and consumers may avoid overeating. Reducing food serving with nudges such as reduced dish weight may have the additional boon of reducing food waste in cases where consumers do not in fact eat the entirety of the portion served.

### 3.3 Limitations and future research

The current research suffers from several limitations that could potentially be addressed by further research. First, the documented effects focused on cases where serving dishes like trays are carried concurrently with serving. In many situations, a tray or plate may be carried right before or after a serving, for instance in cafeterias where diners put their trays down before serving food onto them. In the current studies, participants held the dishes while serving themselves. Thus, the studies only explored immediate, rather than delayed, effects, of carrying heavier weights.

The current research only examines the weight of serving dishes. Future research may examine whether the effects extend to other contexts where consumers do not intend to immediately consume food, such as grocery stores, where carrying heavier baskets, for example, may lead to increased food purchases.

The current studies included fairly small sample sizes, though similar findings were obtained across the studies, supporting the validity and reliability of the findings. Further, as recruiting and managing experiments with actual food items and consumption is costly and timely, a trade off exists with sample size. Accordingly, prior research efforts involving actual eating behavior often used even smaller samples than those used in the current studies [e.g., 67–69]. Despite the fact that such sample sizes and smaller ones are fairly common in studying actual food consumption behavior, future research should conduct large replications, particularly in field settings, to further support the reliability and validity of the findings.

Since the current studies focused on the weight of trays and plates, further studies may explore the effects of other types of weights consumers might carry while serving food. For example, the weight of other serving dishes (bowls, cups). Along similar lines, the current studies demonstrate effects on a limited array of foods. Future studies could add to the generalizability of the current findings by demonstrating effects for other types of foods and by varying the cultures studied, given likeability of foods, as well as which foods are considered healthy, may vary by culture.

In addition, it may be interesting to explore the nature of the relationship between the underestimation of weight carried and actual weight carried (as found in study 2). Studies could also explore whether serving greater amounts of caloric foods when carrying extra weight constitutes a form of functional behavior, a way to drive people to fulfill their higher energetic needs given weight carried. It may be that when carrying more weight, one has greater energy needs. When need is greater, any amount of food is lower relative to one’s needs, which may lead to reduced judgments of quantity as well as serving greater amounts of food. This effect may be additive to that of reduced sensitivity to weight.

## 4. Conclusion

Consumers tend to serve themselves greater amounts of food when carrying heavier serving dishes. This is because reduced sensitivity to the weight of foods served can lead consumers to continue serving more food without being aware of the increases in food served. Carrying heavier weights reduces consumers’ sensitivity to the amount of food served. Without a good sense of the amount of food served, consumers may not know when to stop serving. They may then continue serving more food than they would with lighter serving dishes, especially when serving well liked foods. On a practical level, the findings suggest it may be a good idea to use lighter dishes when serving food—lightening plates may in turn lighten stomachs.

## Supporting information

S1 Appendix (DOCX)
