# Supplementary material for: Weighing heavy: Heavy serving dishes increase food serving
Source: PLoS One. 2023 Aug 25;18(8):e0288956. doi: 10.1371/journal.pone.0288956 (PMC10456203; doi:10.1371/journal.pone.0288956)
Supplement: S1 Appendix — (DOCX) [file pone.0288956.s001.docx]

Appendix

## Study 3: Extension to Likable (Unhealthy) Choices

Studies 1 and 2 showed that heavier serving dishes led consumers to serve themselves more food. Specifically, the studies offered evidence for increases in amounts of food served, and especially for foods that are well liked. In other words, serving dish weight seems to selectively nudge consumers towards increased serving, or choice, of well-liked foods.

The current study aims to extend the finding of the prior studies in multiple ways. First, it aims to apply a different serving dish – a plate. Second, given the important role of liking in the process of reduced sensitivity, the current study builds on the idea that if consumers serve themselves more when they are less sensitive to the amount of food served (under the heavy serving dish condition) they may also increase unhealthy choices, given unhealthy food items are more likable. This is because the reduced sensitivity to the weight of food served allows consumers to indulge more in well-liked foods, without the potential guilt from serving more of well-liked but potentially less healthy foods. Given the results of studies 1 and 2, then, one can predict a tendency to gravitate towards an increase in well liked, and less healthy items, which may result in less healthy choices. This leads to the following prediction:

**H_4_: Heavier serving dish weight leads consumers to make less healthy choices.**

- 1. *Methods*

*Pretest.* A pre-test was conducted as a manipulation check for plate weight, to determine whether chosen plate modifications sufficed to create differences in the perceived weight. Forty undergraduate students (32 female) participated in a one-factor (heavy versus light plate) between-subjects design. Participants were asked to hold the plate and assess its weight. A set of especially designed plates was used. The real weights of the plates were 1,152 grams for the heavy plate and 745 grams for the light one. As expected, a clear difference was observed in estimated weights: *Mheavy* = 1,252 grams, *Mlight* = 491grams, *t*(38) = 2.596, *p* = .013.

*Main study.* Fifty-four undergraduate students (44 female) participated in a lab study involving a one-factor (heavy versus light plate) between-subjects design. The lab mimicked a shopping environment with multiple units of 10 choice pairs (e.g., granola bar versus chocolate bar, cucumber versus tomato) set up on different tables (their order was changed each hour; controlling for order did not change the nature of the reported results). Participants were given a plate (either heavy or light) by the experimenter and, one at a time, went around the room selecting whichever options they preferred from each choice pair. In 3 of the 10 food choice pairs, the options involved equally healthy or unhealthy pairs (banana versus apple, cucumber versus tomato, and two types of salty snacks). These choices were included to mask the main pairs of interest —7 of the 10 choice pairs— in which one option was healthier than the other based on a pre-test (white-wheat roll versus whole-wheat roll, chocolate crackers versus whole wheat crackers, chocolate yogurt versus plain yogurt, chocolate covered orange snack versus carrot snack, high fat cheese versus low fat cheese, and chocolate candies versus raisins). The dependent variable was the number of unhealthy choices made. For the seven choice pairs of interest, an unhealthy food choice was coded as ‘1’ whereas a healthy food choice was coded as ‘0’; thus, the dependent variable score ranged from 0 to 7 where the maximum reflects a choice of 7 unhealthy food items and the minimum reflects a decision to choose 7 healthy food items. In addition, participants were asked to estimate the total number of calories of the snacks they put on their plate.

### Results and Discussion

The results of study 3 based on ANOVA indicate that participants in the heavy plate condition were more likely to make unhealthy choices. Specifically, the analysis reveals that the difference in number of unhealthy food choices between the heavy and the light conditions was significant (45.9% difference): *Mheavy* = 2.64, *Mlight* = 1.81; *F*(1,52) = 4.59, *p* = .037, *η _p_^2^* = .08. Further and similarly to the findings in study 2, participants in the heavy plate condition estimated a marginally lower number of calories compared to the participants in the light plate condition (2,164.62 vs. 3,054.35, t_47_=1.55, p=0.064).

Overall, the current study extends the link between weight of the serving dish —in this case of a plate— and actual food choices. The results suggest that heavier plates lead to stronger preferences for unhealthy food choices, supporting H_4_. This finding is in line with the notion that heavier serving dishes appear to lower consumers’ sensitivity to the food they serve, the calories they consume, and the choices they make, especially for foods people like, which are often unhealthy foods.
